# Incorporating epigenetic data into the risk assessment process for the toxic metals arsenic, cadmium, chromium, lead, and mercury: strategies and challenges

**DOI:** 10.3389/fgene.2014.00201

**Published:** 2014-07-16

**Authors:** Paul D. Ray, Andrew Yosim, Rebecca C. Fry

**Affiliations:** ^1^Department of Environmental Sciences and Engineering, Gillings School of Global Public Health, University of North CarolinaChapel Hill, NC, USA; ^2^Curriculum in Toxicology, School of Medicine, University of North CarolinaChapel Hill, NC, USA

**Keywords:** risk assessment, DNA methylation, miRNA, histone modification, disease, epigenomics, epigenetics

## Abstract

Exposure to toxic metals poses a serious human health hazard based on ubiquitous environmental presence, the extent of exposure, and the toxicity and disease states associated with exposure. This global health issue warrants accurate and reliable models derived from the risk assessment process to predict disease risk in populations. There has been considerable interest recently in the impact of environmental toxicants such as toxic metals on the epigenome. Epigenetic modifications are alterations to an individual's genome without a change in the DNA sequence, and include, but are not limited to, three commonly studied alterations: DNA methylation, histone modification, and non-coding RNA expression. Given the role of epigenetic alterations in regulating gene and thus protein expression, there is the potential for the integration of toxic metal-induced epigenetic alterations as informative factors in the risk assessment process. In the present review, epigenetic alterations induced by five high priority toxic metals/metalloids are prioritized for analysis and their possible inclusion into the risk assessment process is discussed.

## Introduction

### Risk assessment

The US Environmental Protection Agency (EPA) conducts human health risk assessments for toxic chemicals using a four tier process as part of the Integrated Risk Information System (IRIS) Program (EPA, 2014a). This four step process includes hazard identification, dose-response assessment, exposure assessment, and risk characterization (EPA, 2014b). At its core, the aim of the risk assessment process is to determine whether exposure to a particular agent has the potential to induce deleterious health outcomes, and to quantify the risk associated with various exposures. In order to conduct such an assessment, the chemical/agent's mode and mechanism of action are ideally determined, as well as the toxicokinetics (TK) and toxicodynamics (TD) associated with the chemical agent (EPA, 2007).

The human health risk assessment process begins by first defining the individuals or populations at risk, the sources and routes of exposure, associated adverse health outcomes, and any cofounding variables which may affect analyses of exposure or disease state (EPA, 1992). Once these factors have been defined, the process of hazard identification can be initiated. Hazard identification aims to determine whether a particular agent may increase the incidence of specific adverse health outcomes, and whether such outcomes are likely in humans (EPA, 2012a). Here any relevant data from human clinical or epidemiological studies are used to determine if there are associations between an agent and adverse health outcomes (EPA, 2009). Utilizing both human *in vivo* and *in vitro* models, the mode of action for a particular agent is investigated (reviewed in Clewell, 2005; Crump, 2011), as well as the toxicokinetic and toxicodynamic profiles (Heinrich-Hirsch et al., 2001). However, if little or no data exists for a particular agent or environmental toxicant in humans, animal studies will be reviewed and assessed but must statistically account for uncertainties arising from utilizing animal models in place of human data (Dellarco and Baetcke, 2005).

The next step of the human health risk assessment process is the dose-response assessment. The dose-response assessment seeks to determine a relationship between exposure to an agent and the severity and incidence of adverse health effects (EPA, 2014a,b). First, data are analyzed to identify ranges of exposure or dose and correlated with changes in adverse health outcomes, as well as any mechanisms of action associated with the adverse health outcomes. Again, where human data are scarce, data from rodent or primate models will be utilized. It is often rare that ample data are available for a particular agent to cover the range of exposures being studied. In these cases, extrapolation is used to estimate health outcomes for particular exposures. It is at this stage that modeling of the dose-response of a particular agent has included uncertainty factors (UFs) (Stedeford et al., 2007). UFs have been utilized to compensate for limited data, including data obtained from animal models and variability between human populations. These UFs may be multiplied together, and used alongside measurements of effect such as the benchmark dose (BMD) and benchmark dose lower confidence limit (BMDL), no observed adverse effect level (NOAEL), or lowest observed adverse effect level (LOAEL) to determine the reference dose (RfD) (EPA, 1993), or reference concentration (RfC). The RfD and RfC are calculated as an estimated level of daily oral and inhalation exposure that present no increased lifetime risk of negative health effects from a particular agent. Lastly, a dose-response relationship is determined by utilizing the analyzed data to determine linearity between exposure to the agent and disease or mechanism of action; furthermore, any correlations between dose or exposure and the mode of action can be utilized to determine if there are biological threshold values (EPA, 2011, 2012b).

Following the dose-response assessment, an exposure assessment is conducted to quantify an individual or population's exposure (EPA, 2011). The intent of the exposure assessment is to estimate or measure the duration, frequency, and extent of human exposure to the agent under conditions to which the individual or population is likely to be exposed (EPA, 2014b). While hazard identification and dose-response assessments provide associations between an agent and adverse health outcomes, quantification of exposure is necessary to formulate and classify the overall risk of the agent. For example, an extremely carcinogenic agent may not pose a health risk if the opportunity for exposure to the agent is insignificant or non-existent (EPA, 2005a). These data are collected to quantify the types of exposure, length of exposure, frequency, and intake route, to include absorption, distribution, and excretion of the agent (EPA, 1992). This information is then utilized both in the risk characterization step, as well as a means of determining vulnerable populations, such as children, whom may experience higher exposure or may be highly susceptible to the agent (EPA, 2005b).

The final step in the human health risk assessment process is risk characterization. The risk characterization is based upon the type and degree of risk, and consists of an integrative analysis of the summarized findings of each of the previous assessments with their associated uncertainties (EPA, 2000). The compiled analyses are used to make recommendations concerning the risk an agent may pose to the population, and such recommendations may be utilized to drive policy making and form regulations (Schmidt, 2004).

### Epigenetic data in the risk assessment process

Environmental contaminants have the potential to mediate disease states through perturbations in key signaling pathways via differential gene expression. Such perturbation can ultimately result in altered protein expression and activity. In the last two decades, research has shown that epigenetic alterations, a term describing the processes that govern heritable alterations in genomic expression that are not dependent upon changes in the DNA sequence (Cortessis et al., 2012), play a major role in the transcriptional processes that regulate gene expression. Epigenetic alterations regulate homeostatic and inducible gene expression and include but are not limited to DNA methylation, the post-translational modifications of histones, and small non-coding RNAs (Weake and Workman, 2010). The field of toxico-epigenomics, which is the study of the relationship between epigenetic alterations and adverse cellular outcomes in response to toxic agents, is now at the forefront of the field of environmental health science. It is possible that the epigenetic alterations associated with exposure to environmental toxicants may be key factor in the etiology of environmentally-associated diseases (Dolinoy and Jirtle, 2008; Baccarelli and Bollati, 2009; Haluskova, 2010; Hou et al., 2012). However, the role/potential utility of epigenetic data in the risk assessment process has yet to be defined.

The interest in utilizing toxico-epigenomic data builds upon the history of the desire for the incorporation of toxico-genomic data in the risk assessment process. Toxico-genomics, the study of the relationship between the products of the genome, mRNA and protein, and the cellular response to toxic insult, garnered much interest with the advent of high throughput technologies developed in the late, 1990s such as the gene expression microarray. Genome-wide profiling of transcriptional responses as a consequence of exposure to toxicants has allowed for the harnessing of the predictive value of mRNA transcripts in relation to the pathogenesis and progression of environmental contaminant-induced disease (Aardema and MacGregor, 2002). Gene expression profiling has been considered a potential tool in the risk assessment process (Pennie et al., 2004; Bourdon et al., 2013; Euling et al., 2013), based on the principles that toxicity induces changes in gene expression patterns, adverse outcomes in response to toxicity are a result of differential gene expression, and gene expression in response to toxicity is sensitive and expression patterns may serve as biomarkers (Aardema and MacGregor, 2002). The identification of transcriptional events that mediate pathogenic processes allows those events to serve as biomarkers in the risk assessment process. Several investigations have utilized gene profiling to predict biological outcomes in response to contaminant exposure (Wang et al., 2005). Transcript profiles have also been correlated with associated biological pathways, such as inflammation and oxidative stress (Scandalios, 2005; Beaulieu et al., 2014).

If adverse health outcomes can be predicted based upon gene expression profiles, then factors controlling gene expression may also be used to predict these same outcomes. Epigenetic data may therefore be used to inform the risk assessment process given the epigenetic regulation of gene expression in response to toxicants.

There are several benefits of using epigenetic data as determinants in the risk assessment process. For example, epigenetic alterations can be used as biomarkers of effect upon exposure to environmental toxicants. These epigenetic biomarkers may also be employed as predictors of disease when such epigenetic marks are associated with differential gene expression. There is also the potential that such alterations may be heritable and therefore stable in the context of detection. They may also predict or demonstrate possible inherited gene expression changes in response to maternal toxicant exposure (as reviewed in Ho et al., 2012). As a result, there is the potential that epigenomic data may ultimately inform TD, TK, the inter- and intra-species differences in TD and TK, mechanisms of action, mode of action, and contribute to the exposure and dose-response assessment. Ultimately, such toxico-epigenomic data may be useful in the risk characterization process and add additional accuracy to the risk assessment process.

In order to incorporate epigenetic data in the risk assessment process, the following parameters must be addressed: are toxicant-induced epigenetic alterations dose responsive, are the changes toxicant specific, are the modifications genome-wide or gene specific, and are the modifications accurate predictors of biological endpoints? In the context of epigenetic data that could inform human health risk assessment, an overview of epigenetic alterations in the context of five priority toxic metals and their relationship to gene expression and disease is presented.

### Epigenetics and gene expression changes

Epigenetic alterations regulate key events in cellular homeostasis, including transcriptional and translational regulation of gene expression. The most well studied epigenetic alterations are DNA methylation, the covalent, post-translational modification of histones, and non-coding small RNAs (miRNA). Epigenetic alterations can be induced by environmental stimuli, and much attention has been given to the role of the epigenome in human disease (Skinner, 2011); a role that arises primarily from the control the epigenome exerts over the transcriptome and proteome.

**Figure 1:**
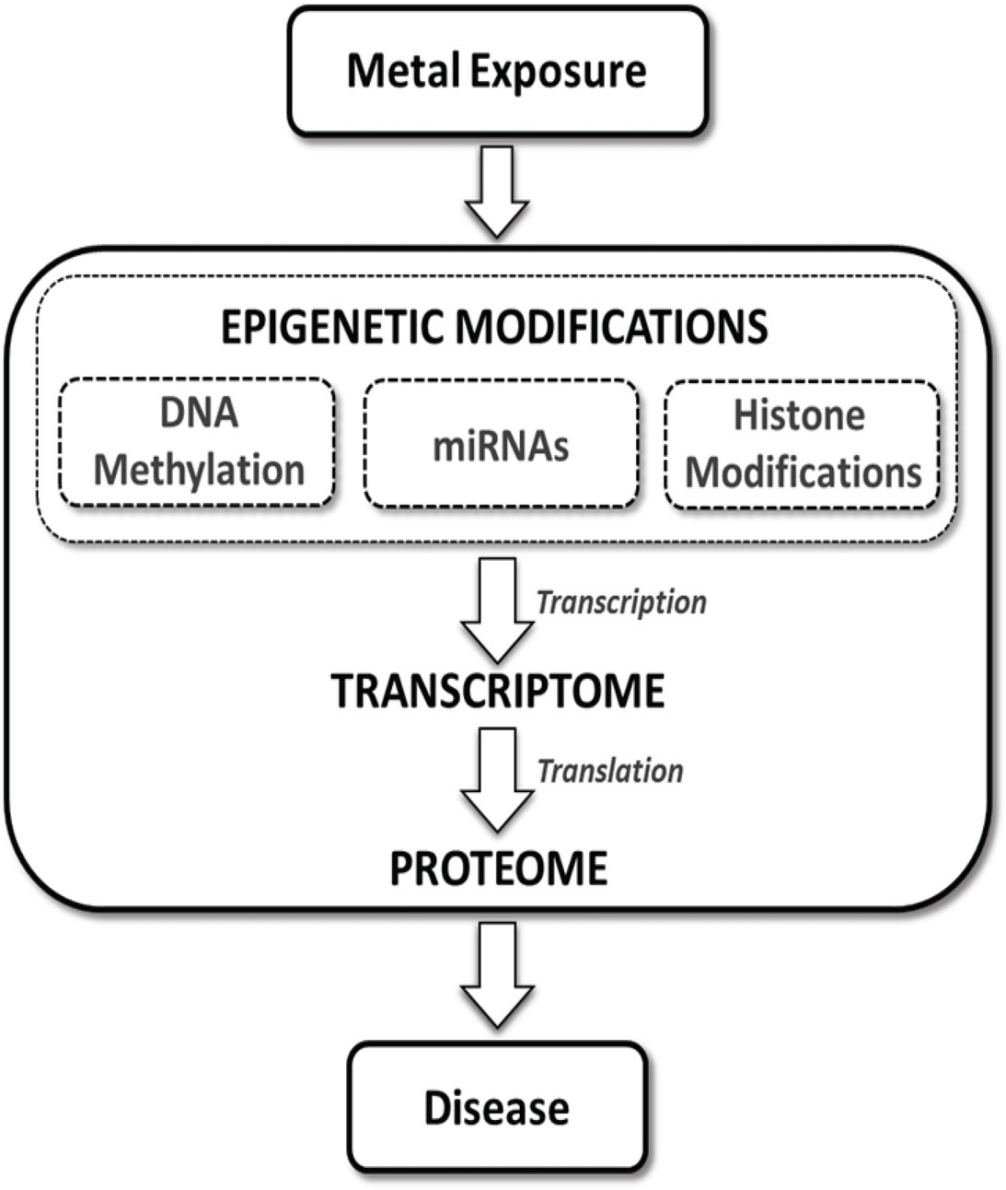
**The role of the epigenome in toxic metal-induced disease pathways**. Epigenetic alterations classified broadly as effects to the “epigenome” have the potential to regulate mRNAs/transcripts (i.e., the transcriptome) and ultimately impact protein expression (i.e., the proteome) within cells. Exposure to toxic metals can impact various components of the epigenetic machinery. These toxic metal-mediated epigenetic alterations may directly impact gene transcription and subsequently regulate protein translation, leading to aberrant expression of key mediators of disease processes.

The transcriptome, which is the total transcribed RNA of a cell at a given point in time, is regulated primarily through transcription and mRNA stability and degradation. DNA methylation, histone modifications, and miRNA all regulate the transcriptome through transcriptional processes. The proteome, the total protein of the cell, is the functional mediator between the genome and the cell and is regulated primarily through post-transcriptional regulation of mRNA transcripts (Figure 1). Epigenetic regulation of the proteome occurs primarily through the action of miRNAs on mRNA transcript stability and translation.

#### The epigenetic event of DNA methylation

DNA methylation is the most well-studied epigenetic alteration. The transfer of a methyl group to cytosine forms 5-methyl cytosine (5-MeC) which occurs preferentially, though not exclusively, on cytosines in CpG dinucleotides. The DNA methyltransferase (DNMT) family mediates the methylation of cytosines through the transfer of a single methyl group from S-adenosine methionine (SAM) to cytosine. At present, there has been no identification of a single enzyme which exclusively demethylates DNA (Brocato and Costa, 2013).

It is believed that CpG methylation silences genes by preventing access of transcription factors to their respective binding sites either directly by methylation of transcription factor binding sequences, or through the recruitment of methyl-CpG-binding domain (MBD) proteins. MBD proteins may directly block transcription factor binding or recruit chromatin modifiers that alter the chromatin structure into a more transcriptionally repressive environment (Newell-Price et al., 2000). In regulating gene transcription, DNA methylation has been implicated in disease. DNA methylation is associated with cancer and in some cases has proven to be a viable biomarker for disease. For example, DNA hypermethylation of the glutathione S-transferase pi 1 (*GSTP1*) gene was correlated with prostate cancer (Esteller et al., 1998), thus serving as a diagnostic tool, while hypermethylation of O 6-methylguanine-DNA methyltransferase (*MGMT*) aids in decisions for therapeutic strategies for glioma (Esteller et al., 2000). In addition to cancer, DNA methylation is associated with Alzheimer's disease, diabetes, atherosclerosis, Friedrich's ataxia, immunodeficiency, rheumatoid arthritis, multiple sclerosis, and systemic lupus erythematosus (Heyn and Esteller, 2012).

#### The epigenetic event of histone modification

To accommodate the size of the genome, DNA is packaged into chromatin. Chromatin may be partitioned into condensed regions known as heterochromatin, or more “relaxed,” accessible regions called euchromatin (Felsenfeld and Groudine, 2003). Any cellular function involving DNA is inherently affected by the chromatin structure; gene regulation, DNA replication, repair, condensation, and segregation are all highly regulated by chromatin remodeling (Weake and Workman, 2010), which is the modulation of the chromatin structure. In gene transcription for example, large transcriptional components must have access to binding elements within the gene promoter, and so chromatin remodeling is necessary to procure a “relaxed” state to allow access. The central component of chromatin is the nucleosome, consisting of 147 bp of DNA wrapped ~1.7 superhelical turns around an octamer of the core histone proteins H2A, H2B, H3, and H4 (Kinner et al., 2008). Histones share the presence of an N-terminal tail that can be post-translationally modified (Kinner et al., 2008). It is in part through the post-translational, covalent modification of histones that chromatin remodeling is mediated.

There are several distinct classes of histone modifications; lysine acetylation, methylation, ubiquitylation, and sumoylation; arginine methylation and deamination; proline isomerization and glutamate poly-ADP ribosylation; and lastly, serine and threonine phosphorylation (Wang et al., 2007). Histone modifications are mediated by histone acetyltransferases (HAT), histone deacetylases (HDAC), serine and threonine kinases, phosphatases, and histone methyltransferases and demethylases (Berger, 2007). In addition to the complexity enabled by multiple histone modifications, single histone modifications can offer even more complexity by the mono, di, or even tri-methylation of a single lysine residue or the symmetrical or asymmetrical di-methylation of arginines (Berger, 2007).

Histone modifications regulate transcription through a variety of means, from directly disrupting DNA-nucleosome contacts, to recruiting and binding non-histone proteins and additional histone modifiers. Histone modifications may disrupt contact between DNA and histones through histone lysine acetylation and methylation, resulting in “relaxed” and condensed chromatin states, respectively, which are generally correlated with transcriptional activation and repression (Reinke and Horz, 2003; Shogren-Knaak et al., 2006; Grewal and Jia, 2007). However, the disruption of DNA-histone contact is not mediated solely by the direct effect of a single (or multiple) histone modification, but in conjunction with the recruitment of non-histone proteins to the modified histone residue. Histone modifications recruit non-histone proteins through specific binding domains. Through these binding domains, non-histone effectors bind histone modifications, resulting in further chromatin remodeling. Several aspects of the transcriptional process are governed by histone modifications, including enhancer element activation, accessibility of the transcription start site, RNA polymerase recruitment, transcriptional initiation, and others beyond the scope of this review (Weake and Workman, 2010; Gardner et al., 2011).

Altered histone modifications have the potential to impact differential gene and subsequent protein expression. Differential histone modifications have therefore been implicated in several diseases, including cancers, and non-cancer endpoints such as Parkinson's and Huntington's disease (Heyn and Esteller, 2012). Interestingly, other diseases associated with differential histone modification such as Alzheimer's disease, diabetes, atherosclerosis, Friedrich's ataxia, and systemic lupus erythematosus, are also associated with aberrant DNA methylation (Heyn and Esteller, 2012).

#### The epigenetic event of miRNA signaling

miRNAs are small, non-coding RNAs ~21–23 nucleotides in length that post-transcriptionally regulate gene expression through complementary binding of 3′ or 5′ untranslated regions (3′ or 5′ UTR) of mRNA. miRNA target the transcripts for degradation or to prevent translation, resulting in decreased target protein expression, though it has been reported that miRNAs may activate translation (Vasudevan et al., 2007; Breving and Esquela-Kerscher, 2010). miRNAs may also regulate transcription by directly binding to gene promoter sequences and inducing chromatin remodeling (Zardo et al., 2012).

miRNA sequences are located in specifically associated promoters, or even embedded in host genes (Breving and Esquela-Kerscher, 2010). After transcription miRNAs are processed in the nucleus to form hair-pin structures known as pre-miRNA. Pre-miRNA are then exported into the cytoplasm where they undergo further processing into mature miRNA. Mature miRNA involved in gene silencing are incorporated into RNA-induced silencing complexes (RISC) (Palanichamy and Rao, 2014). At present, over 17,000 distinct mature miRNAs have been identified in over 140 species (Kozomara and Griffiths-Jones, 2011).

Aberrant expression of miRNAs has been associated with several human disease states, including cancer, cardiovascular disease, and genetic disorders (Mendell and Olson, 2012). miRNAs could be readily employed as biomarkers of exposure and disease due to the availability of circulating extracellular miRNAs found in body fluids, such as amniotic fluid, saliva, serum, and plasma. Additionally, circulating miRNAs have been shown to be correlated with cardiovascular disease and tumors (Mendell and Olson, 2012), suggesting a strong association between detectable levels and disease.

### Incorporating metals-associated epigenetic data into the risk assessment process

In the present review, epigenetic alterations associated with exposure to the five toxic metals/metalloids inorganic arsenic, cadmium, chromium, lead, and mercury are summarized. These data are examined in order to propose various ways in which the epigenetic data may be integrated into the human health risk assessments process. The selection of these prioritized toxic metals for this review was based on the 2011 ASTDR Substances Priority List where they rank in the top five (ATSDR, 2011). In so far as it is possible, the present review limited studies to human populations or human cell-derived results in order to maximize the relevance of the data to risk assessment. While the studies embodied in this review are by no means completely comprehensive, the attempt was to reflect the current state of epigenetic studies in the literature. In Tables 1–**5** studies that have examined metals-associated epigenetic alterations, including those that were assessed at a functional level, are summarized.

**Table 1:** **Epigenetic alterations induced by inorganic arsenic (iAs) or iAs metabolites**.

**Class**	**Cell lines or biological samples (location)**	**Assessment/modification**	**Identified gene targets**	**Dose (range)**	**References**
DNA methylation	A549 (human lung carcinoma cells)	Global and targeted/hypermethlation	*TP53*	0–10 μM NaAsO_2_, 0–300 μM KH_2_AsO_4_, 0–2000 μM DMA	Mass and Wang, 1997
DNA methylation	Caco-2 (human colon cancer cells)	Global and targeted/hypermethylation	*TP53*	0, 1, or 2 μmol/L As^3+^ for 7 d	Davis et al., 2000
DNA methylation	UOK (human kidney cell line) A549 (human lung carcinoma)	Targeted/hypermethylation	–	0.08–2 μM NaAsO_2_ or 30–300 μM NaH_2_AsO_4_	Zhong and Mass, 2001
DNA methylation	RWPE-1 (immortalized human prostate epithelial cells)	Global/hypomethylation	–	5 μM NaAsO_2_	Benbrahim-Tallaa et al., 2005
DNA methylation	Peripheral blood leukocytes (West Bengal, India, *n* = 158)	Targeted/hypermethylation	*TP53*	<50 to >500 μg iAs in DW for >6 mo	Chanda et al., 2006
			*TP16*	
DNA methylation	HepG2 (human liver cancer cell line)	Targeted/hypermethylation	↓ TP16^*^	2–10 μM of As_2_O_3_	Cui et al., 2006b
			↓ CDH1^*^		
			↓ RASSF1A^*^		
			↓ GSTP1^*^		
DNA methylation	Bladder tumors (New Hampshire, USA, *n* = 351)	Targeted/hypermethylation	*RASSF1A*	95% percentile: =0.26 μg/g As	Marsit et al., 2006a
			*PRSS3*		
DNA methylation	SV-HUC-1 (normal human urothelial cell line)	Targeted/hypermethylation	↓ DAPK^*^	2, 4, and 10 μM NaAsO_2_	Chai et al., 2007
DNA methylation	Uroepithelial carcinoma tumor specimens (Taiwan, *n* = 38)	Targeted/hypermethylation	*DAPK*		Chen et al., 2007
DNA methylation	Peripheral blood leukocytes (Dhaka, Bangladesh, *n* = 294)	Global/hypermethylation	–	0.1–860 μg/L As	Pilsner et al., 2007
DNA methylation	HaCat (immortalized human keratinocytes)	Global/hypomethylation	–	0.2 μM As^3+^	Reichard et al., 2007
DNA methylation	Peripheral blood leukocytes (Guizhou, China, *n* = 170)	Targeted/hypermethylation	↓ TP16^*^		Zhang et al., 2007
DNA methylation	RWPE-1 (immortalized human prostate epithelial cells)	Targeted/hypomethylation	–	5 μM As^3+^ for up to 16 wk	Coppin et al., 2008
DNA methylation	UROtsa (immortalized human urothelial cells)	Global/differentially methylated	–	1 μM As (III) or 50 nM MMA (III)	Jensen et al., 2009a
DNA methylation	Peripheral blood leukocytes (Dhaka, Bangladesh, *n* = 386)	Global/hypermethylation	–	26–208 μg/L As	Pilsner et al., 2009b
DNA methylation	Molt4, MUTZ-1, U937, U266 and CA46 (malignant hematological cell lines)	Targeted/hypomethylation	↑ CDKN2B^*^	0.5, 1.0, or 2.0 μM As_2_O_3_	Fu et al., 2010
			↑ CDKN2A^*^		
DNA methylation	Peripheral blood leukocytes (West Bengal, India, *n* = 64)	Global/hypermethylation	–	<50 to >500 μg iAs in DW for >6 mo	Majumdar et al., 2010
DNA methylation	SV-HUC-1 (normal human urothelial cell line)	Targeted/hypermethylation	↑ RECK^*^	1, 4, or 10 μM NaAsO_2_	Huang et al., 2011
DNA methylation	Peripheral blood leukocytes (Zimapan, Mexico, *n* = 16)	Genome-wide/hypermethylation	181 *DMGs*	7–77 μg/g Creatinine	Smeester et al., 2011
DNA methylation	Peripheral blood leukocytes (Matlab, Bangladesh, *n* = 101)	Global/male: hyper female: hypo	–	127.6 μg/L mean urinary arsenic	Pilsner et al., 2012
DNA methylation	Peripheral blood leukocytes (Zimapan, Mexico, *n* = 16)	Genome-wide/hypomethylation	812 *DMGs*	3.6–31.8 ng As/ml tAs in urine	Bailey et al., 2013
DNA methylation	Peripheral blood leukocytes (New Hampshire, USA, *n* = 134)	Genome-wide/hypermethylation	68,353 *DM CpG loci*	0.03–100 μg/L DW As	Koestler et al., 2013
DNA methylation	Peripheral blood mononuclear cells (Bangladesh, *n* = 320)	Global/hypermethylation	–	0–300+ μM/L DW As	Niedzwiecki et al., 2013
DNA methylation	Peripheral blood leukocytes (Bangladesh, *n* = 44)	Genome-wide/hypermethylation	71 *DMGs*	Median = 12 μM/L in DW	Kile et al., 2014
DNA methylation	HepG2 and HEK-293 (human liver cancer cell line and human embryonic cell line) (Murshidabad, West Bengal, India, *n* = 245)	Targeted/hypomethylation	↑ ERCC2^*^	0–10 μM As(III)	Paul et al., 2014
DNA methylation	Human urothelial carcinoma tumors (Southwestern Taiwan, *n* = 28)	Genome-wide/hypermethylation (majority)	*213 DMGs*	0.25–20.08 ppm yr As	Yang et al., 2014
miRNA	TK-6 (immortalized human lymphoblast cell line)	Genome-wide/	–	2 μM NaAsO_2_ for 6 d	Marsit et al., 2006b
		↓ miR-210			
		↑ miR-22			
		↑ miR-34a			
		↑ miR-221			
		↑ miR-222			
miRNA	T24 (human bladder carcinoma)	Targeted/	↓ PTEN^*^	4 μM As_2_O_3_	Cao et al., 2011
		↓ miR-19a			
		↑ miR-222			
miRNA	Hep-G2 (human hepatocellular carcinoma)	Genome-wide/	–	4 μM As_2_O_3_	Meng et al., 2011
		↑ miR-24			
		↑ miR29a			
		↑ miR30a			
		↑ miR-210			
miRNA	Newborn cord blood (Gómez Palacio, Mexico, *n* = 40)	Genome-wide/	–	6.2–319.7 μg/L U-tAs	Rager et al., 2014
		↑ let-7a			
		↑ miR-107			
		↑ miR-126			
		↑ miR-16			
		↑ miR-17^*^			
		↑ miR-195			
		↑ miR-20a			
		↑ miR-20b			
		↑ miR-26b			
		↑ miR-454			
		↑ miR-96			
		↑ miR-98			
Histone modification	WI-38 (human diploid fibroblast)	↑ H3S10p	↑ C-Jun^*^	400 μM As^3+^ for 10, 30, 60 min	Li et al., 2003
			↑ C-Fos^*^		
Histone modification	CGL-2 (hybrid of the HeLa adenocarcinoma cell line and normal human fibroblasts)	↑ H2AXp	–	0–10 μM As^3+^ for 24 h	Yih et al., 2005
Histone modification	RPMI7951 (human malignant melanoma epithelial-like cell line)	↑ H2AXp	–	2.5 μM As^3+^ for 24 h	Zykova et al., 2006
Histone modification	UROtsa (human urothelium non-tumorigenic cell line)	↑ H3ac	↑ DBC1^*^	1 μM As^3+^ (chronic)	Jensen et al., 2008
		↑ H3K27me3	↓ FAM83A^*^		
		↑ H3K4me2	↓ ZSCAN12^*^		
		↑ H3K9me2	↓ C1QTNF6^*^		
			↑ NEFL^*^		
			–		
Histone modification	Hep-G2 (human hepatocarcinoma line)	↑ H3K9ac	–	7.5, 10, 15, and 50 μM NaAsO_2_ for 2, 4, 12, or 24 h	Ramirez et al., 2008
Histone modification	UROtsa (human bladder epithelial cells)	↓ H4K16ac	–	1, 3, 10 μM NaAsO_2_ or MMA^III^O at 0.3, 1, 3 μM	Jo et al., 2009
Histone modification	Human lung carcinoma cells (A549)	↑ H3K4me3	-	1 μM As^3+^ for 24 h	Zhou et al., 2009
Histone modification	Peripheral blood Leukocytes (Brescia, Italy *n* = 63)	↑ H3K4me2	–	0.01–0.31 μg/m^3^ arsenic PM for 3 d	Cantone et al., 2011
		↑ H3K9ac			
Histone modification	Urine (Bangladesh, *n* = 40)	↑ H3K9me2	–	91.5 μg/L urinary arsenic	Chervona et al., 2012
		↓ H3K9Ac			
		H3K4me3			
		H3K27me3			
		H3K27Ac			
		H3K18Ac			
Histone modification	HaCaT (human keratinocytes)	↑ H4R3me2	↑ PRMT1^*^	10 μM NaAsO_2_ for 2, 4, 8, 12, 24 h	Huang et al., 2013
		↑ H3R17me2	↑ PRMT4^*^		
Histone modification	UROtsa (human bladder epithelial cells)	↓ H3K9me2	↑ WNT5A^*^	1 μM As^3+^ (chronic)	Jensen et al., 2009b
		↓ H3K27me3			
		↑ H3Ac			

↑, increased; ↓, decreased;

* , functionally validated at the expression level; –, not functionally validated at the expression level; Global refers to global methylation patterns; Genome-wide refers to high throughput gene-specific assays; DMGs, differentially methylated genes.

## Metals and epigenetic alterations

### Arsenic exposure and associated epigenetic alterations

Inorganic arsenic an environmentally ubiquitous toxic metalloid (henceforth referred to as a metal) contaminating drinking water around the globe. Chronic exposure through contaminated drinking water constitutes a major health concern, and it is estimated that hundreds of millions of individuals are exposed, a large percentage of which are estimated to exceed the World Health Organization (WHO) drinking water limit of 10 ppb (Nordstrom, 2002; WHO, 2006; Centeno et al., 2007).

While a significant research emphasis has been placed upon the reduction of chronic exposure to arsenic, it is estimated that millions of individuals continue to be exposed in Bangladesh alone (Flanagan et al., 2012). Using Bangladesh as a case study, inorganic arsenic is predicted to contribute significantly to the global health burden, based on its associated contribution of over 17,000 disability-adjusted life years (DALYs) and over 9000 deaths attributed to arsenic-related disease in the country (Lokuge et al., 2004).

Acute exposure to inorganic arsenic is associated with health endpoints that include encephalopathy and peripheral neuropathy (Ratnaike, 2003), and chronic exposure is implicated in cardiovascular, reproductive, respiratory, neurological, diabetic, and gastrointestinal disorders (WHO, 2006; Das and Sengupta, 2008; Sengupta et al., 2008; Pruss-Ustun et al., 2011). Inorganic arsenic is classified as a Group 1 human carcinogen (IARC, 1987, 2004) and is associated with kidney, skin, urinary, liver, and lung cancers (IARC, 2004; WHO, 2006; Straif et al., 2009). Proposed mechanisms of action in arsenic-mediated carcinogenesis are alterations in cellular signaling pathways, perturbations in the DNA damage response and repair pathways, chromosomal aberrations, and oxidative stress (Gebel, 2001; Hughes, 2002; Kligerman and Tennant, 2007; Kitchin and Wallace, 2008; Kitchin and Conolly, 2010).

It is also becoming increasing clear that epigenetic alterations may play a role in arsenic toxicity and disease. Exposure to inorganic arsenic has been associated with epigenetic modifications such as changes in global and gene specific DNA methylation, histone acetylation, methylation, and phosphorylation, and altered expression of miRNAs. These alterations induced by inorganic arsenic may contribute to carcinogenesis and non-cancer endpoints (reviewed in Ren et al., 2011; Bailey and Fry, 2014b). While further study is needed to clearly define the role of epigenetic alterations in arsenic-associated toxicity and disease, progress may be made in determining the application of these data to the risk assessment process utilizing the available epigenetic data. Studies relating inorganic arsenic exposure and epigenetic modifications are summarized in Table 1.

#### Arsenic-associated changes in DNA methylation

DNA methylation is the most commonly studied epigenetic alteration induced by inorganic arsenic. In humans, inorganic arsenic is biotransformed into its methylated forms; the metabolism process converts trivalent arsenite (iAs^III^) and pentavalent arsenate (iAs^V^) to trivalent and pentavalent methylated forms. The primary enzyme involved in the required reduction and methylation reactions is arsenic methyltransferase (AS3MT), which utilizes SAM as a methyl donor (Thomas et al., 2004, 2007; Drobna et al., 2009). DNA methylation and biotransformation of iAs both employ SAM as a methyl donor. While a trend of hypomethylation is seen, the effect of arsenic-induced SAM depletion on DNA methylation is inconclusive (Zhao et al., 1997; Reichard et al., 2007; Coppin et al., 2008; Nohara et al., 2011). The activity of DNMT, the primary enzyme mediating DNA methylation, is generally decreased upon exposure to inorganic arsenic, though the exact mechanisms are unknown (Zhao et al., 1997; Cui et al., 2006b; Fu et al., 2010). The majority of studies report that arsenic induces global DNA hypomethylation; however, instances have been reported of arsenic inducing global hypermethylation (Mass and Wang, 1997; Davis et al., 2000). Of note is that biotransformation of iAs is not essential for DNA hypomethylation (Coppin et al., 2008).

Genome-wide DNA hypermethylation was observed in peripheral blood leukocytes from arsenic exposed individuals in India and Bangladesh (Pilsner et al., 2007, 2009b; Majumdar et al., 2010). DNA hypermethylation has been observed in a dose-dependent manner, but only in the presence of adequate folate (Pilsner et al., 2007). Interestingly, increased risk of arsenic-related skin lesions was associated with DNA hypomethylation (Pilsner et al., 2009b). This is important as global hypomethylation has been implicated in carcinogenesis (Eden et al., 2003; Wilson et al., 2007). In addition to genome-wide DNA methylation, gene specific DNA methylation in human populations has also been demonstrated. DNA methylation of the tumor suppressor cyclin-dependent kinase inhibitor 2A (*CDKN2A*/*p16*) promoter has been positively associated with arsenic exposure in individuals from West Bengal, India (Chanda et al., 2006) and Guizhou, China (Zhang et al., 2007). However, no relationship between arsenic exposure and methylation of the *CDKN2A*/*p16* promoter was found in a study conducted in New Hampshire (Marsit et al., 2006a). In urothelial carcinomas, promoter methylation of tumor suppressors protease serine 3 (Homo sapiens; *PRSS3*) and Ras association (RalGDS/AF-6) domain family member 1 (*RASSF1A*) (Marsit et al., 2006a) and death-associated protein kinase 1 (*DAPK*) (Chen et al., 2007) was correlated with arsenic exposure between individuals exposed and not exposed from New Hampshire and Taiwan.

Arsenic exposure alters DNA methylation of genes involved in tumorigenesis (reviewed in Bailey and Fry, 2014a). Tumor suppressor genes that are methylated in response to arsenic exposure include *CDKN2A* (Davis et al., 2000), tumor protein 53 (*TP53*) (Mass and Wang, 1997; Davis et al., 2000), von Hippel–Lindau tumor suppressor (*VHL*) (Zhong and Mass, 2001), *RASSF1A* (Cui et al., 2006a), *DAPK* (Chai et al., 2007), and reversion-inducing-cysteine-rich protein with kazal motifs (*RECK*) (Huang et al., 2011). DNA methylation of oncogenes in response to arsenic include cyclin D1 (*CCND1*) (Chen et al., 2004), estrogen receptor alpha (*ER-α*) (Chen et al., 2004; Waalkes et al., 2004) and members of the RAS family of small G-proteins such as *HRAS* and *KRAS* (Benbrahim-Tallaa et al., 2005). Our group identified 183 differentially methylated promoters associated with arsenic exposure in adult subjects from Zimapan, Hildago, Mexico (Smeester et al., 2011), and out of this group were 17 tumor suppressor or tumor suppressor-associated genes with hypermethylated promoters.

As seen in human populations, *in vitro* studies also demonstrate inorganic arsenic-induced changes in DNA methylation status of promoter regions. For example, arsenic trioxide treatment of malignant cell lines decreased hypermethylation of *RASSF1A*, *p16, GSTP1* (Cui et al., 2006b), and cyclin dependent kinase 2A/2B (*CDKN2A/CDKN2B*; *p16/p15*) (Fu et al., 2010) promoters and concomitantly increased expression of mRNA levels of these genes. Increased arsenic exposure induces DNA hyper- and hypomethylation of regions of the genome relative to unexposed cells in human kidney and lung cell lines (Zhong and Mass, 2001), and in arsenic transformed human urothelial cell lines (Jensen et al., 2009a). Also of interest to risk assessment is that focal DNA methylation patterns are stable events that exist in arsenic-transformed cells even after arsenic exposure has ceased (Jensen et al., 2009a). Similar observations of correlations between focal DNA methylation and gene expression have been reported in iAs^III^- and MMA^III^-transformed human urothelial cells. An inverse relationship between mRNA levels and DNA hypermethylation of promoters was reported for C1q and tumor necrosis factor related protein 6 (*C1QTNF6*), deleted in bladder cancer 1 (*DBC1*), G0/G1switch 2 (*G0S2*), family with sequence similarity 83, member A (*FAM83A*), keratin 7 (*KRT7*), zinc finger and SCAN domain containing 12 (*ZSCAN12*), epiregulin (*EREG*), and thioesterase superfamily member 4 (*THEM4*) (Jensen et al., 2008, 2009a).

Proper development of the fetus is dependent upon establishment of fetal DNA methylation patterns, which can influence the health of individuals even into adulthood (reviewed in Kim et al., 2009). Alterations in DNA methylation patterns associated with *in utero* exposure may influence the expression of genes/proteins involved in key signaling pathways that underlie adverse health outcomes associated with gestational arsenic exposure. Thus, the study of alterations in DNA methylation profiles in response to gestational arsenic exposure is an area of great interest.

#### Arsenic-associated changes in histone modifications

Chervona et al. demonstrated that in individuals exposed to inorganic arsenic in Bangladesh, total urinary arsenic was inversely correlated with global H3K9Ac and positively correlated with H3K9me2 (Chervona and Costa, 2012). In addition gender specific differences were found with a positive correlation between well water arsenic and global H3K4me3 and H3K27me3 in females and a negative correlation between males (Chervona et al., 2012), demonstrating the viability of associating arsenic exposure and histone modifications in human populations. An additional study showed that histone modifications were associated with arsenic in steel workers exposed to particulate matter (PM) containing arsenic. A positive correlation was reported between H3K4me2 and H3K9Ac and cumulative exposure to arsenic and nickel-containing PM in peripheral blood lymphocytes (PBL) (Cantone et al., 2011). Given the broad range of histone modifications induced by inorganic arsenic in cultured human cell lines, further exploration of histone modifications in humans exposed to arsenic is warranted. Exploration of the relationship between arsenic-induced histone modifications and diseases associated with chronic arsenic exposure would be of great value for risk assessment. The majority of histone modifications associated with arsenic exposure, however, has been demonstrated *in vitro*.

Huang et al. demonstrated that arsenic induces the expression of the anti-oxidant gene ferritin through methylation of H4 arginine 3 (H4R3) and H3 arginine 17 (H3R17) in a dose-dependent manner in human keratinocytes (Huang et al., 2013). Transient knockdown of the methyltransferases PRMT1 and PRMT4 resulted in decreased histone methylation and ferritin expression; most interesting was the decrease in the transcription factor Nrf2 binding to the ferritin promoter (Huang et al., 2013). This suggests that arsenic induces transcription factor recruitment and promoter binding through histone methylation. Arsenic is also a potent inducer of H3S10 phosphorylation (H3S10p), and this event has been associated with up-regulation of genes involved in tumorigenesis (Li et al., 2003). Ablation of H3S10p through chemical inhibition, kinase knockdown, or the use of a transfected histone H3S10A mutant results in decreased cell transformation in several models (Choi et al., 2005, 2008a; Zippo et al., 2007; Kim et al., 2008). These data suggest a possible association between arsenic exposure, histone phosphorylation, and carcinogenesis. At extremely high concentrations, inorganic arsenic induces a DNA damage response, and so was found to induce H2AX phosphorylation in the context of DNA damage and apoptosis (Yih et al., 2005; Zykova et al., 2006).

Arsenic exposure induces global and focal histone acetylation and methylation in human cell lines (Ramirez et al., 2008; Zhou et al., 2008, 2009; Jo et al., 2009; Chu et al., 2011). Arsenite modulates global H4K16Ac (Ramirez et al., 2008; Jo et al., 2009) as well as heterochromatic-specific H3K4me2 and euchromatic-specific H3K4me3 increases (Zhou et al., 2009). Histone acetylation by arsenic is also gene specific; H3 phosphoacetylation by arsenic was associated with upregulation of caspase 10, apoptosis-related cysteine peptidase (*CASP10*), and the proto-oncogenes *C-Jun* and *C-Fos* (Li et al., 2002, 2003). Differential H3 acetylation of several promoters was reported in arsenic transformed urothelial cells compared to untransformed cells (Jensen et al., 2008). mRNA expression of the hypoacetylated *FAM83A, DBC1, C1QTNF6*, and *ZSCAN12* genes, as well as the hyperacetylated neurofilament, light polypeptide (*NEFL*) gene was analyzed; there was a positive correlation between mRNA levels and H3 acetylation (Jensen et al., 2008). Crosstalk between histone modifications and DNA methylation has been reported, as a correlation was found between DNA hypermethylation and H3 hypoacetylation at the promoters of FAM83A, ZSCAN12, C1QTNF6, and DBC1 (Jensen et al., 2008, 2009a). Another study demonstrated that histone modifications play a major role in gene expression implicated in As-mediated carcinogenesis (Jensen et al., 2009b). WNT5A was identified as a key player in arsenic-induced malignant transformation of human urothelial cells. In turn, *WNT5A* expression was regulated by permissive and repressive histone modifications, most notably H3K27 methylation and H3 acetylation. The latter was shown to be stable as it was maintained after malignant transformation and withdrawal of arsenic exposure (Jensen et al., 2009b).

Several studies have demonstrated the mechanisms of arsenic-induced histone modifications, focusing on the expression and activity of histone modifying enzymes. Increases in H3K9 acetylation by arsenic in hepatocellular carcinoma cells was correlated with inhibition of HDAC activity (Ramirez et al., 2008). In human lung A549 cells, arsenic was shown to increase H3K9 di- and tri-methylation through increased expression of the H3K9 methyltransferase G9a; however there was no change in expression of lysine (K)-specific demethylase 3A (JHDM2A), the major demethylase involved in H3K9 dimethylation (Zhou et al., 2008). These studies demonstrate the broad range of histone modifications induced by inorganic arsenic exposure in the context of the expression of genes associated with arsenic-mediated diseases. There is the possibility that these genes represent possible biomarkers of inorganic arsenic-induced disease.

#### Arsenic-associated changes in miRNA expression

In human lymphoblasts, arsenic altered the expression of five miRNAs; miR-221, miR-222, miR-210, miR-34a, and miR-22 (Marsit et al., 2006b). The biological roles of miRNAs in cells exposed to arsenic have also been investigated. miR-29a was associated with arsenic-mediated apoptosis in hepatocellular carcinoma cells (Meng et al., 2011) as well as miR-19a in human bladder carcinoma cells (Cao et al., 2011).

Malignant transformation of *TP53*^−/−^ cells in response to arsenic treatment was correlated with decreased levels of miR200b (Wang et al., 2011). Decreased expression of miR200b occurred concomitantly with methylation of the miR-200 promoter, suggesting that arsenic may transcriptionally repress miRNA expression through DNA methylation (Wang et al., 2011). These studies demonstrate the role of specific miRNAs in arsenic-mediated tumorigenesis, thus serving as possible key biomarkers for disease risk.

#### Epigenetic data in the arsenic risk assessment process

As a specific example demonstrating how arsenic-associated epigenetic modifications may inform the risk assessment process, we considered DNA methylation patterning in response to arsenic exposure. Specifically this was considered in the context of the second step in the risk assessment process, namely the dose-response assessment. A study of Bangladeshi adults with chronic exposure to arsenic via contaminated drinking water demonstrated dose-responsive global hypermethylation of DNA in peripheral blood leukocytes (Pilsner et al., 2007). In a separate study, dose-responsive DNA hypermethylation of the tumor suppressors *TP53* and *TP16* was observed among individuals with chronic exposure to arsenic in West Bengal, India compared with control individuals (Chanda et al., 2006). These findings imply that DNA methylation observed in these studies is dose responsive with arsenic exposure and thus may help to inform the dose-response assessment step. In combination with studies that have found associations between arsenic-induced DNA methylation patterns and negative health endpoints (as reviewed in Tseng, 2007), these findings may also ultimately aid in informing the hazard identification step as well. The integration of dose-dependent changes in epigenetic profiling with functional changes in gene/protein expression will be needed to fill in knowledge gaps related to the dose-dependent relationships.

### Mercury exposure and associated epigenetic alterations

Mercury is ranked third by the ATSDR (2011) Substance Priority List (ATSDR, 2011) and is listed as one of the WHO's top 10 chemicals of major public health concern (WHO, 2003). This ubiquitous element is found in the earth's crust and is released into the air by natural geological phenomena such as volcanic eruptions and oceanic evaporation following weathering of the crust. Humans are primarily exposed to mercury through occupational exposure including metal smelting, gold mining, coal burning, electric industries, and wood production; dietary exposure from seafood; and ambient air exposure from the burning of fossil fuels and evaporation from mercury contaminated waste (reviewed in Boening, 2000; Tchounwou et al., 2003; Spiegel, 2009). It is estimated that as developing countries make the transition to heavy industrialization, increasing levels of mercury will be introduced into the air, which may lead to greater inhalation and dietary exposure (Zhang et al., 2002; UNEP, 2013).

Mercury occurs in a variety of forms including the elemental form, inorganic mercury, and organic mercury salts, which includes the highly toxic methylmercury (MeHg). Although exposure differs dramatically based on location, occupation, and diet, the 2009 National Health and Nutrition Examination Study (NHANES), estimates that ~2% of American women of reproductive age exceed the oral RfD for mercury (EPA, 2013). This is particularly disconcerting, as a number of studies have shown that MeHg readily crosses the placenta, and *in-utero* exposure is associated with a host of negative health outcomes including learning disabilities, reduced cognitive function, immune suppression, and a host of neurological disorders (reviewed in Zahir et al., 2005; Holmes et al., 2009; Park and Zheng, 2012). In children and adults, mercury exposure has been associated with negative health outcomes in cardiovascular systems, respiratory systems, neurological systems, as well as changes in the thyroid, liver, kidney, and immune function (reviewed in Counter and Buchanan, 2004; Holmes et al., 2009). In addition to exposure of vulnerable populations such as children and the developing fetus, acute and chronic exposure is predicted to afflict untold numbers of individuals worldwide, and continues to contribute to the global burden of disease. To date, few human studies have explored the epigenetic alterations induced by mercury. Studies relating exposure to mercury and epigenetic alterations are summarized in Table 2.

**Table 2:** **Epigenetic alterations induced by mercury**.

**Epigenetic modification**	**Cell lines or biological samples (location)**	**Assessment/modification**	**Identified gene targets**	**Dose (range)**	**References**
DNA methylation	Blood (San Francisco, USA, *n* = 58)	Targeted/hypermethylation	*GSTM1*	Blood Hg: 2.9 μg/L	Hanna et al., 2012
DNA methylation	Buccal mucosa samples (Michigan, USA, *n* = 131)	Targeted/hypomethylation	*SEPP1*	Hair Hg: 0.31–0.44 (μg/g)	Goodrich et al., 2013
				Urine Hg: 0.60–0.83 (μg/L)	
mi-RNA	NT2 (carcinoma pluripotent stem cells)	Genome-wide/	–	400 nM MeHgCl for 2–36 d	Pallocca et al., 2013
		↑ miR-302b			
		↑ miR-367			
		↑ miR-372			
		↑ miR-196b			
		↑ miR-141			

↑, increased; ↓, decreased; ^*^, functionally validated at the expression level; –, not functionally validated at the expression level; Global refers to global methylation patterns; Genome-wide refers to high throughput gene-specific assays; DMGs, differentially methylated genes.

#### Mercury-associated changes in DNA methylation

Currently, few human studies have investigated changes in DNA methylation patterns related to environmental mercury exposure. One study found increased methylation of the promoter region of glutathione S-transferase mu 1 (*GSTM1*) in women with elevated blood mercury levels (above 2.9 μg/L). However, no statistical correlation was found and the study did not ascertain *GSTM1* expression levels (Hanna et al., 2012). Another study measured mercury exposure in hair samples and found that for males there was a correlation between DNA hypomethylation of the selenoprotein P plasma 1 (*SEPP1*) promoter and increasing mercury levels (Goodrich et al., 2013). As in the former study, expression levels of *SEPP1* were not investigated. Both genes in these two studies are involved in the cellular antioxidant response system, which mitigates oxidative stress. Though gene expression was not measured in these two cases, there is the potential that DNA methylation of the promoters would influence their expression and thus alter the oxidative stress response.

#### Mercury-associated changes in histone modifications

At present, the majority of investigations into histone modification resulting from exposure to environmental mercury or its compounds have been conducted in non-human studies. In mouse models, exposure to MeHg was associated with increased H3K27 trimethylation (H3K27m3) along with decreased histone H3 acetylation in the promoter region of brain derived neurotrophic factor (*BDNF*) (Onishchenko et al., 2008). Another group investigating mouse embryonic stem cells found exposure to mercury chloride (HgCl_2_) decreased total histone protein (THP) and was associated with decreased H3K27 mono-methylation (Gadhia et al., 2012). Given the conservation of cellular responses in mice to humans and the effect of other metals on histone modifications in humans, it may be predicted that mercury also induces histone modification in humans.

#### Mercury-associated changes in miRNA expression

In carcinoma pluripotent stem cells, exposure to methyl mercury chloride (MeHgCl) was associated with increased expression of miR-302b, miR-367, miR-372, miR-196b, and miR-141 (Pallocca et al., 2013). It was shown that these miRNAs were associated with developmental processes and cellular responses to stress, and pathway analysis on possible mRNA targets revealed possible links to neurological development including learning and memory formation.

#### Epigenetic data in the mercury risk assessment process

The few human studies investigating epigenetic alterations related to mercury exposure may be considered as examples of epigenetic alterations useful to inform the risk assessment process. Specifically, it was demonstrated that increasing mercury levels were correlated with DNA hypomethylation of the *SEPP1* promoter (Goodrich et al., 2013); these data provide a foundation that could eventually inform the dose-response step of the risk assessment process. Correlations between metal-responsive epigenetic alterations and signaling pathways involved in disease could also inform the hazard identification step. For example, it was demonstrated that mercury-mediated induction of miRNAs may be involved in the regulation of pathways involved in stress response (Pallocca et al., 2013). Still, additional data are needed to provide causal links between miRNA induction by metals with metal-mediated alterations in cellular stress response signaling pathways. This provides a starting point by which information on miRNA modulation may be introduced into the hazard identification step of the risk assessment process.

### Lead exposure and associated epigenetic alterations

Lead is a naturally occurring element that is widely known for its human health impacts. Low levels of exposure has been primarily associated with adverse effects in the nervous (Finkelstein et al., 1998), immune (Mishra, 2009), cardiovascular (Navas-Acien et al., 2007), and renal systems (Kim et al., 1996), as well as a reduction in male fertility (Sallmen, 2001), negative pregnancy outcomes (Juberg et al., 1997; Xie et al., 2013), behavior problems (Bellinger et al., 1994), impaired cognitive function (Canfield et al., 2003), lowered IQ scores (Nevin, 2000; Canfield et al., 2003), and violence (Stretesky and Lynch, 2001; Olympio et al., 2009). These effects have been associated with both chronic and acute exposure, and have been linked to exposure in the womb or during childhood (reviewed in Flora, 2002). Known primarily for effects in the neurological development of children, prenatal and childhood exposure to lead has been shown to reduce cognitive function at both high (Bellinger et al., 1987) and low doses (reviewed in Lanphear et al., 2005; Jedrychowski et al., 2009). Exposure to the metal or its salts primarily occurs through inhalation or ingestion (reviewed in Tong et al., 2000; Moreira and Moreira, 2004).

Considerable public health action has been undertaken in the regulation of lead in gasoline (Nriagu, 1990), paint (Farfel and Chisolm, 1990), and piping (Wong and Berrang, 1976) that has been effective in reducing blood lead levels (BLLs) in the United States (Pirkle et al., 1994; Muntner et al., 2005). However, exposure to lead continues to be a problem both in the United States and worldwide (Warniment et al., 2010), with almost a half million children exceeding the Center for Disease Control and Prevention (CDC) level of concern of 5 μl/dl (CDC, 2013), although at present there is no safe BLL (CDC, 2012). Worldwide, the poisonous element is estimated to contribute almost 0.6% to the global burden of disease, and as of 2000, an estimated 240 million individuals had BLLs above 5 μl/dl (WHO, 2000). While the mechanism of action for lead's long term neurological effects are still not completely understood, it is suspected that the formation of reactive oxygen species, altered gene expression, and changes to calcium signaling may play a role in the pathogenesis of lead-associated disease (reviewed in Sanders et al., 2009). Studies relating lead exposure and epigenetic modifications are summarized in Table 3.

**Table 3:** **Epigenetic alterations induced by lead**.

**Class**	**Cell lines or biological samples (location)**	**Assessment/modification**	**Identified gene targets**	**Dose (range)**	**References**
DNA methylation	Maternal Tibia (Mexico, *n* = 103)	Global/hypomethylation	–	10.5 ± 8.4 (μg/g)	Pilsner et al., 2009a
DNA methylation	Peripheral blood leukocytes (Greece, *n* = 19)	Targeted/hypermethylation	*TP16*	6–100 (ug/dL)	Kovatsi et al., 2010
DNA methylation	Patella (USA, *n* = 2280)	Global/hypomethylation	–	27.4 ± 19.7 (g/g)	Wright et al., 2010
DNA methylation	Peripheral blood leukocytes (San Francisco, California, *n* = 41)	Targeted/hypomethylation	*COL1A2*	0.3–8.8 (μg/L)	Hanna et al., 2012
DNA methylation	A431 (epidermoid carcinoma cells)	Targeted/hypomethylation	↑ COX-2^*^	0.1–10 μM for 0.5–2 h	Tsai et al., 2014
mi-RNA	Peripheral blood leukocytes	Targeted/	-	3 d lead PM	Bollati et al., 2010
		↑ miR-222			
	(Brescia, Italy, *n* = 63)	↓ miR-146a			

↑, increased; ↓, decreased;

* , functionally validated at the expression level; –, not functionally validated at the expression level; Global refers to global methylation patterns; Genome-wide refers to high throughput gene-specific assays; DMGs, differentially methylated genes.

#### Lead-associated changes in DNA methylation

To date, few studies have been conducted to assess associations between DNA methylation and lead exposure. The studies that have been conducted tend to focus on measures of global genomic methylation, such as measuring Alu or long interspersed nuclear elements-1 (LINE-1) (Yang et al., 2004). In one study, maternal tibia lead levels were shown to have an inverse association with methylation of Alu and LINE-1 in newborns (Pilsner et al., 2009a). No association was noted between cord BLLs of methylation of Alu and Line-1. However, in a separate study conducted with an older cohort, tibia lead levels were not correlated with LINE-1 or Alu methylation, and were not predictive of global methylation levels (Wright et al., 2010). The same study did find patella lead levels had an inverse correlation with methylation of LINE-1, but not Alu methylation. Based on these results, it is theorized that historical exposure captured in the patella is associated with decreased methylation of LINE-1 elements, whereas more recent exposure captured in the tibia is associated with decreased methylation of both LINE-1 and Alu in infants (Wright et al., 2010). In the context of the early life exposures, such results imply that the one of the most vulnerable populations, developing fetuses, may experience changes in global methylation as a result of historical maternal lead exposure and accumulation.

In women seeking *in vitro* fertilization (IVF), there was an inverse association between lead exposure and methylation of the promoter region of the gene Collagen Type I, Alpha 2 (*COL1A2*) (Hanna et al., 2012). In a separate study, individuals with high blood lead concentrations had methylation of the tumor suppressor, *CDKN2A/TP16*, whereas individuals with low blood lead concentrations only had partial methylation of *TP16* (Kovatsi et al., 2010). In another study, cells from the carcinoma cell line A431 were exposed to lead ions (Pb2+) which lead to a reduction in methylation of the gene prostaglandin-endoperoxide synthase 2 (*PTGS2*) (Tsai et al., 2014).

At present, additional research is needed to detail the mechanisms underlying the relationship between lead exposure and altered levels of DNA methylation. It has been theorized that the generation of reactive oxygen species may inhibit the binding of methyl-CpG binding proteins and ultimately change the functionality of DNA methyltransferase (Pilsner et al., 2009a). Additionally, exposure to lead in *in vitro* models of human neuroblastoma cells inhibited the activity of IGF-1, which acts directly on methionine synthase (Waly et al., 2004). Methionine synthase is one of many enzymes crucial in the regulation of DNA methylation, and disruption of normal enzymatic levels by inhibition of IGF-1 may present another possible pathway linking lead exposure and methylation. While addition research is needed, it is clear that individuals, children, and developing fetuses may experience global, or site specific methylation in response to environmental lead exposure. In the event that these changes in DNA methylation are associated with functional changes in gene and protein expression, they may present a viable mode of action to explain some of lead's toxicity and related negative health outcomes.

#### Lead-associated changes in histone modifications

Currently, to our knowledge, no human studies have investigated histone modification resulting from exposure to environmental lead. However, researchers studying primate models have found a reduction in proteins associated with histone modification linked to infant lead exposure (Bihaqi et al., 2011).

#### Lead-associated changes in miRNA expression

As with histone modification, the field of miRNA changes as a result of environmental lead exposure is sparse. At present, only one study has investigated changes in miRNA expression in response to lead. The study, conducted on peripheral blood leukocytes exposed via inhalation, found miR-222 expression showed a positive association with lead exposure, while miR-146a expression was negatively correlated with lead exposure (Bollati et al., 2010). miR-146a, which was down regulated, has been shown to be associated with inflammation in rodent models (Boldin et al., 2011), and is predicted to play a role in the innate immune response (Taganov et al., 2006). miR-222, which had increased expression due to lead exposure has been shown to regulate the tumor suppressor, Cyclin-dependent kinase inhibitor 1B (p27Kip1), and down regulation of p26Kip1 has been linked to increased cell proliferation and higher incidences of various cancers (Le Sage et al., 2007).

#### Epigenetic data in the lead risk assessment process

While the adverse health effects of high dose lead exposure have been known for some time, studies have found that previously labeled “safe” levels of lead may be associated with negative health outcomes (Lanphear et al., 2005; Zhang et al., 2013), thus necessitating additional lead risk assessments. The use of data on lead-mediated epigenetic alterations may help to inform the hazard identification step of the lead risk assessment process. One study demonstrated that lead exposure as measured by maternal tibia and patella samples, was correlated with global DNA hypomethylation in umbilical cord blood (Pilsner et al., 2009a). A separate study demonstrated in a cohort of older individuals that patella lead levels were associated with global DNA hypomethylation (Wright et al., 2010). Such findings can aid in determining the risk of fetal adverse health outcomes in response to maternal lead exposure. These studies also increase the understanding of lead's mechanisms of action, and may ultimately be useful as alternative measures for historical or cumulative biomarkers of exposure. These results may help to inform the hazard identification process particularly if the results can be linked to health endpoints resulting from decreased global DNA methylation, especially during embryonic development. Ultimately, such results may help to link additional health outcomes to current biologically relevant exposures.

### Cadmium exposure and associated epigenetic alterations

Cadmium is an environmental contaminant associated with considerable global disease burden. Exposure to cadmium occurs primarily through cigarette smoke and dietary sources (reviewed in ATSDR, 2008; Jarup and Akesson, 2009). Present in almost all foods, levels of cadmium vary greatly depending upon the type of food and source contamination; high concentrations are found in crustaceans, mollusks, and offal meats such as the liver and kidney. However, plant matter such as leafy green vegetables and root vegetables contain higher cadmium levels than do meat (Jarup and Akesson, 2009). Cadmium has a long half-life in humans; specifically 10–30 years in the kidney, which is the main target organ of cadmium toxicity in response to chronic dietary exposure (Godt et al., 2006). In addition to renal toxicity, cadmium exerts toxicity on bone, and is associated with osteoporosis and osteomalacia (“Itai Itai” disease of Japan). Unlike other toxic metals, cadmium is a partial transplacental agent and accumulates in the placenta and has been implicated in adverse birth outcomes (Kippler et al., 2012).

Cadmium is classified as a Group I carcinogen, associated with cancers of the liver, bone, kidney, and pancreas as well as a known risk factor for cardiovascular disease (reviewed in ATSDR, 2008; Jarup and Akesson, 2009; Tellez-Plaza et al., 2012). Our lab has demonstrated that low dose cadmium exposure in human lymphoblast cells alters the expression of genes involved in various biological functions including cancer and cardiovascular disease (Benton et al., 2011). Suggested mechanisms of action of cadmium exposure include inhibition of DNA repair, generation of reactive oxygen species, and perturbation of cell cycle progression or apoptosis (Waisberg et al., 2003). As with other toxic metals prioritized in this review, the effect of cadmium on gene expression has been studied, while the impact of cadmium on changes to epigenetic machinery is largely under studied. Given the exposure levels and resulting toxicity, it is increasingly clear that studies detailing the epigenetic effects of cadmium on toxicity and disease are warranted. Studies relating cadmium exposure and epigenetic alterations are summarized in Table 4.

**Table 4:** **Epigenetic alterations induced by cadmium**.

**Epigenetic modification**	**Cell lines or biological samples (location)**	**Assessment/modification**	**Identified gene targets**	**Dose (range)**	**References**
DNA methylation	RWPE-1 (human prostate epithelial cells)	Global and targeted/hypermethylation	↓ TP16^*^	10 μM CdCl_2_ for 10 wk	Benbrahim-Tallaa et al., 2007
			↓ RASSF1A^*^		
DNA methylation	K562 (chronic myelogenous leukemia cells)	Global/hypomethylation	–	2.0 μM for 24/48 h	Huang et al., 2008
DNA methylation	HLF (human embryo lung fibroblast cells)	Global/hypermethylation	–	0–1.5 μM for 2 mo	Jiang et al., 2008
DNA methylation	JEG-3 (human choriocarcinoma cells)	Targeted/hypomethylation	↑ HSD11B2^*^	0.5, 1 μM Cd^2+^ for 24 h	Ronco et al., 2010
DNA methylation	RWPE-1 (human prostate epithelial cells)	Global/differential methylation	–	10 μM Cd^2^ for 1 yr	Severson et al., 2012
DNA methylation	UROtsa (human urothelial cells)	Global/differential methylation	–	1 μM Cd^2^ for 1 yr	Severson et al., 2012
DNA methylation	16HBE (human bronchial epithelial cells)	Global and Targeted/hypermethylation	↓ hMSH2^*^	5–15 μM CdCl_2_ for 3–4 mos	Zhou et al., 2012
			↓ ERCC1^*^		
			↓ XRCC1^*^		
			↓ hOGG1^*^		
DNA methylation	Maternal venous blood and newborn cord blood (Durham, NC, *n* = 34)	Genome-wide/hyper and hypo methylation	–	0–1.05 μg/L Cd (maternal)	Sanders et al., 2014
miRNA	Peripheral blood leukocytes (Italy, *n* = 63)	Genome-wide/miR-146a	–	0.01 μg/m^3^ 3 d	Bollati et al., 2010
miRNA	HepG2 (human hepatoblastoma cells)	Genome-wide/	–	2, 10 μM CdCl_2_ for 24 h	Fabbri et al., 2012
		↓ let-7 family			
		↓ miR-1233			
		↓ miR-1275			
		↓ miR-130a			
		↓ miR-15b			
		↓ miR-15b^*^			
		↓ miR-23b			
		↓ miR-361-5p			
		↓ miR-455-3p			
miRNA	Human bronchial epithelial cells	Targeted/	↓ CFTR^*^	2 μM CdCl_2_ 24 h	Hassan et al., 2012
		↑ miR-101			
		↑ miR-144			
Histone modification	MCF-7 (human breast adenocarcinoma cells)	↓ H3ac	–	5 μg/mL CdTeQD for 4, 24 h	Choi et al., 2008b
Histone modification	UROtsa (human urothelial cells)	↑ H3K4me3	*MT3*	1 μM CdCl_2_ ~60 d	Somji et al., 2011
		↑ H3K9me3			
		↑ H3K27me3			
Histone modification	*In vitro* kinase assay	↓ H3p	–	1–25 μM CdCl_2_	Barcia-Sanjurjo et al., 2013

↑, increased; ↓, decreased;

* , functionally validated at the expression level; –, not functionally validated at the expression level; Global refers to global methylation patterns; Genome-wide refers to high throughput gene-specific assays; DMGs, differentially methylated genes.

#### Cadmium-associated changes in DNA methylation

In humans, prenatal cadmium exposure has been associated with altered DNA methylation in blood leukocytes (Sanders et al., 2014). These patterns of DNA methylation displayed an enrichment for binding sites of specific transcription factors and were thus hypothesized to represent an “environmental footprint” of transcription factor occupancy during times of DNA methylation (Sanders et al., 2014).

Several *in vitro* studies have correlated changes in DNA methylation and features of the carcinogenesis process in response to cadmium. Differential DNA methylation profiles in immortalized cells lines transformed by chronic cadmium exposure were also seen in established cancer cell lines as well as primary tumor tissues (Severson et al., 2012). These results suggest that cadmium-associated cancers may share DNA methylation signatures and these signatures may serve as biomarkers in the risk assessment process. In the context of cadmium carcinogenesis, it was shown that cadmium induces cell proliferation in K562 cells concomitantly with global DNA hypomethylation. Interestingly, pretreatment with methionine not only abrogated the hypomethylation effect but cell proliferation was well, suggesting that cadmium may induce cell proliferation by decreasing DNA methylation levels (Huang et al., 2008). In cadmium-induced malignant transformation of prostate epithelial cells, DNA hypermethylation was associated with silencing of the tumor suppressor genes *TP16* and *RASSF1A* through cadmium-mediated overexpression of *DNMT3b* (Benbrahim-Tallaa et al., 2007), but not *DNMT1*. However, another study conducted in human lung fibroblasts chronically exposed for a period of 2 months showed that cadmium increased mRNA expression of *DNMT1*, as well as *DNMT3a* and *DNMT3b*, and this corresponded to an increase in global DNA hypermethylation (Jiang et al., 2008). The increases in methyltransferase expression and DNA methylation were dose dependent, and thus may be useful in the risk assessment process. In cadmium-transformed human bronchial epithelial cells, promoter hypermethylation resulted in decreased expression of the DNA repair genes MutS homolog 2 (*MSH2*), excision repair cross-complementation group 1 *(ERCC1)*, X-ray repair complementing defective repair 1 *(XRCC1)*, and 8-oxoguanine DNA glycosylase *(OGG1)* (Zhou et al., 2012). Hypermethylation and gene silencing was associated with overexpression of *DNMT1* and *DNMT3a*. Furthermore, inhibition of DNA methylation reversed the decrease in DNA repair enzyme expression. These three studies collectively demonstrate that cadmium silences tumor suppressor and DNA repair genes through DNA hypermethylation mediated by increased expression of DNA methyltransferases.

Cadmium is a known endocrine disruptor, and in immortalized trophoblasts cadmium increased expression of hydroxysteroid (11-beta) dehydrogenase 2 (*HSD11B2*), which regulates the steroid hormone cortisol. This increase in expression was associated with DNA hypomethylation of the *HSD11B2* gene, suggesting epigenetic mechanisms in cadmium-mediated reproductive toxicity (Ronco et al., 2010).

#### Cadmium-associated changes in histone modifications

Reports of cadmium-induced histone modifications are mainly limited to *in vitro* studies. In cadmium-transformed urothelial cells, levels of H3K4me3, H3K27me3, and H3K9me3 occupancy at the metallothionein 3 (MT3) promoter were increased compared to untransformed cells, suggesting chronic cadmium exposure may alter transcriptional responses through histone modification (Somji et al., 2011). MT3 silenced in untransformed UROtsa cells, was expressed after cadmium transformation and this was mediated by metal transcription factor 1 (MTF1) binding to a metallothionein (MT) metal responsive element (MRE); of special interest was that MTF1 binding was significantly increased in the presence of a HDAC inhibitor, suggesting that histone modification in cadmium-transformed cells is necessary for maximal transcription factor binding and gene activation (Somji et al., 2011). In addition to altering methylation of histone H3 lysine residues, cadmium was shown *in vitro* to decrease H3 auto-phosphorylation by inhibition of the human vaccinia-related kinase VRK1/2 (Barcia-Sanjurjo et al., 2013), demonstrating that cadmium may block histone modifications through inhibition of histone modifying enzymes.

#### Cadmium-associated changes in miRNA expression

Peripheral blood leukocytes from workers at an electric furnace steel plant were analyzed after 3 days of exposure to PM containing cadmium; while the expression of miR-146a in peripheral blood leukocytes was not statistically increased in exposed vs. baseline leukocytes, miR-146a was negatively correlated with occupational cadmium exposure (Bollati et al., 2010). Cadmium has been shown to alter miRNA levels *in vitro*. In a human hepatocellular carcinoma cell line, miRNA expression was analyzed after cadmium exposure; several differentially expressed miRNAs were members of the let-7 family, which exhibits oncogene silencing functions (Fabbri et al., 2012). In studying the role of airway pollutants in cystic fibrosis, Hassan et al. showed that cadmium decreased expression of the cystic fibrosis transmembrane conductance regulator *(CFTR)* in human bronchial epithelial cells (Hassan et al., 2012). *CFTR* is a predicted target of miR-101 and miR-144, and this group further demonstrated that cadmium upregulates the expression of miR-101 and miR-144 (Hassan et al., 2012), suggesting that cadmium, through miRNA induction, may be involved in the pathogenesis of cystic fibrosis. These latter two studies implicate cadmium in two disparate disease states through alterations of miRNA levels and such alterations may be useful in the risk characterization step.

#### Epigenetic data in the cadmium risk assessment process

In relation to the risk assessment process, cadmium has been shown to silence the expression of DNA repair enzymes and tumor suppressor genes in a dose-dependent manner via DNA hypermethylation. Specifically this was associated with an increase in the expression of DNMT family members (Benbrahim-Tallaa et al., 2007; Jiang et al., 2008; Zhou et al., 2012). These data can be applied to the dose-response assessment and hazard identification steps of the risk assessment process, given that silencing of these genes represent key factors in carcinogenesis. DNA methylation induced by cadmium may contribute to the current understanding of cadmium's toxicity as it relates to chronic or acute exposures. Chronic exposure of human cell lines to cadmium induced global DNA hypermethylation (Benbrahim-Tallaa et al., 2007; Jiang et al., 2008; Severson et al., 2012; Zhou et al., 2012); however, acute exposure to cadmium was associated with global DNA hypomethylation in other immortalized cell lines (Huang et al., 2008; Ronco et al., 2010). As both global hyper and hypomethylation are associated with a range of diseases (Robertson, 2005; Wilson et al., 2007), these varied epigenetic effects associated with either acute or chronic exposure may help to elucidate possible mode of action differences between varied exposure paradigms. As a result, such epigenetic data may be employed to predict adverse health outcomes based on length of cadmium exposure.

### Chromium exposure and associated epigenetic alterations

Chromium is utilized in several industrial applications from chrome plating and welding to leather tanning and stainless steel manufacturing (reviewed in Zhitkovich, 2011). However, the bulk of human exposure is mainly through drinking water contaminated through industrial chromium use. Hexavalent chromium (henceforth referred to as chromium except where noted) is highly mutagenic and associated with a wide array of cancers including prostate, bone, leukemia, lymphoma, renal, gastrointestinal, brain, and lung cancer (Langard, 1990). The mutagenic and carcinogenic nature of chromium may be explained in part by the variety of genotoxic lesions that it produces; chromium forms adducts with DNA, induces DNA strand breaks, DNA intra- and interstrand crosslinks, and DNA-protein crosslinking (Shi and Dalal, 1994; Stearns et al., 1995; Zhitkovich, 2005). Chromium also interferes with DNA damage response and repair. A proposed mechanism of action of chromium toxicity is the generation of reactive oxygen species, which are generated during the cellular reduction of hexavalent chromium to intermediate species (Shi and Dalal, 1994). Studies relating exposure to chromium and epigenetic modifications are summarized in Table 5.

**Table 5:** **Epigenetic alterations induced by chromium**.

**Class**	**Cell lines or biological samples (location)**	**Assessment/modification**	**Identified gene targets**	**Dose (range)**	**References**
DNA methylation	Lung cancer tumors (Tokushima, Japan, *n* = 68)	Targeted/hypermethylation	↓TP16^*^	12–38 yr Chromate	Kondo et al., 2006
DNA methylation	Lung cancer tumors (Tokushima, Japan, *n* = 61)	Targeted/hypermethylation	*APC*	12–38 yr chromate	Ali et al., 2011
			*MGMT*		
			↓ hMLH1^*^		
			↓ TP16^*^		
DNA methylation	Red blood cells (Shandong, China, *n* = 175)	Global/hypomethylation	–	0.96–115.01 (μg/L)	Wang et al., 2012
DNA methylation	A549 (human B lymphoblastoid cell line and lung cell line)	Global and targeted/hypomethylation	↑ TP16^*^	5–15 μM K_2_Cr_2_O_7_, 1.25–5 (μg/cm^2^) PbCrO_4_	Lou et al., 2013
Histone modification	CNTRL (human skin fibroblasts)	↑ H2AXp	–	6 μM Cr(VI)	Vilcheck et al., 2006
Histone modification	A549 (human lung carcinoma)	↑ H3K4me (2,3)	↓ MLH1^*^	5–10 μM Cr(VI)	Sun et al., 2009
		↑ H3K9me (2,3)			
Histone modification	A549 (human lung carcinoma)	↑ H3K4me (2,3)	–	0.5–10 μM Cr(VI)	Zhou et al., 2009
miRNA	Blood leukocytes (Brescia, Italy, *n* = 63)	Targeted/	–	3 d PM chromium	Bollati et al., 2010
		↑ miR-222			
miRNA	BEAS-2B (human lung epithelial cells)	Targeted/	–	6 mo exposure to 1 μM Cr(VI)	He et al., 2013
		↓ miR-143			

↑, increased; ↓, decreased;

* , functionally validated at the expression level; –, not functionally validated at the expression level; Global refers to global methylation patterns; Genome-wide refers to high throughput gene-specific assays; DMGs, differentially methylated genes.

#### Chromium-associated changes in DNA methylation

The effect of chromium exposure on DNA methylation in human populations has been documented; in a study investigating the effect of folate deficiency on chromium-induced DNA damage, red blood cells of industrial chromate workers were analyzed and showed that chromium exposure was correlated with global DNA hypomethylation, decreased folate levels, and increased oxidative DNA damage (Wang et al., 2012). DNA methylation alterations in lung tumors have been associated with chromium exposure. Kondo and Takahashi et al. studied the methylation status of tumor suppressor and DNA repair genes in chromium vs. non-chromium lung tumors from workers exposed to chromium; lung tumors of chromium exposed workers showed a correlation between the hypermethylation of *TP16* and human mutL homolog 1 (*hMLH1*) promoters and the silencing of their expression (Takahashi et al., 2005; Kondo et al., 2006). Lung tumors of workers exposed to chromium showed differential DNA hypermethylation of tumor suppressor genes *MGMT* and *APC* promoters as compared to lung tumors of subjects not exposed to chromium (Ali et al., 2011).

In cell culture models, global DNA hypomethylation was induced by chromium in A549 lung cells and B lymphoblastoid cells in the context of cell cycle arrest (Lou et al., 2013). While expression of *TP16*, *CDK4*, and *CDK6* mRNA levels were altered in response to chromium, DNA methylation levels at the *TP16* promoter did not change, suggesting that chromium regulates *TP16* and cell cycle progression independently of promoter specific DNA methylation (Lou et al., 2013). Ding et al. investigated whether DNA methylation regulated repression of E-cadherin in oncogenic transformation, but found that chemical inhibitors of DNA methylation had no effect on E-cadherin expression in lung epithelial cells (Ding et al., 2013). Taken together these data suggest that caution should be taken in interpreting the effect of DNA methylation on chromium regulated expression of genes involved in carcinogenesis given the discrepancy between *in vivo* and *in vitro* studies.

#### Chromium-associated changes in histone modifications

It has been demonstrated *in vitro* that chromium interacts with histone arginine and lysine residues (Levina et al., 2006). Chromium induces phosphorylation of histone H2AX in conjunction with DNA damage (Vilcheck et al., 2006). Chromium may regulate histone biotinylation. In bronchial epithelial cells, hexavalent chromium was shown to decrease expression of biotinidase, an enzyme involved in the histone biotinylation pathway, and this transcriptional event was reversed in the presence of HDAC inhibitors suggesting a role for histone acetylation (Xia et al., 2011). Based on the study, chromium may induce changes in histone modification through altering expression of histone-modifying enzymes. This is further evidenced by Sun et al., who demonstrated in bronchial epithelial cells that chromium increased expression of the H3K9-specific methyltransferase G9a, which was correlated with increases of di and tri-methylated H3K9 as well as H3K4 (Sun et al., 2009). H3K9me occurred not only globally, but also at the promoter of *MLH1*, a DNA mismatch repair enzyme. Chromium exposure decreased expression of MLH1 (Sun et al., 2009), suggesting that chromium silences expression of this gene through H3K9me, providing an epigenetic link between chromium and carcinogenesis. Repression of E-cadherin is a possible mechanism of chromium-mediated oncogenic transformation of lung epithelial cells, and chromium increased binding of HDAC1 to the E-cadherin promoter, suggesting increased histone acetylation as a repressive mechanism, though H3K9Ac status was not confirmed (Ding et al., 2013).

#### Chromium associated changes in miRNA expression

Peripheral blood leukocytes from workers at an electric furnace steel plant were analyzed for metals and miRNA levels; chromium was negatively associated with miR-146a (Bollati et al., 2010). He et al. showed that miR-143 is repressed in chromium-transformed human lung epithelial cells, and this repression activates angiogenesis through an IL-8-insuling growth factor receptor (IGFR)-HIF-1 pathway (He et al., 2013).

#### Epigenetic data in the chromium risk assessment process

As an example of the utilization of chromium associated epigenetic modification data to inform the risk assessment process, chromium has been associated with histone modifications in a dose-dependent manner. After exposure to chromate, A549 cells showed increased H3K9me2 and decreased H3K4me3 in the promoter region of the mismatch repair gene *MLH1* (Sun et al., 2009). Furthermore, the results demonstrated that histone alterations were correlated with decreased expression of *MLH1*. *MLH1* has been shown to be associated with multiple cancer and non-cancer endpoints including hereditary colorectal cancer (Kuismanen et al., 2000; Pokorny et al., 1997). As a result, dose-dependent histone modification data may be useful in the hazard identification and dose-response assessment, and may help to identify the molecular mechanisms linking metals exposure to carcinogenesis or other disease outcomes.

## Discussion

### Integration of epigenetic data into the risk assessment process

The risk assessment process attempts to define associations between specific health outcomes and exposure to a specific agent, and to determine levels of exposure at which negative health outcomes associated with the exposure are minimized. The process is systematic and driven by available data in order to understand the mechanistic links between a range of exposures and adverse health effects.

In the present review, a discussion of the potential use of epigenomic data in the risk assessment process is built upon strategies aimed at inclusion of genomic data in the risk assessment process (EPA, 2002). Epigenomic data has the potential to inform both mechanisms and modes of action and in combination with genomic data may identify novel modes of action. Epigenomic data may also be used to identify toxicodynamic (TD) and toxicokinetic (TK) data, inter- and intraspecies differences in TD and TK, exposure assessments, and dose-response assessments.

**Figure 2:**
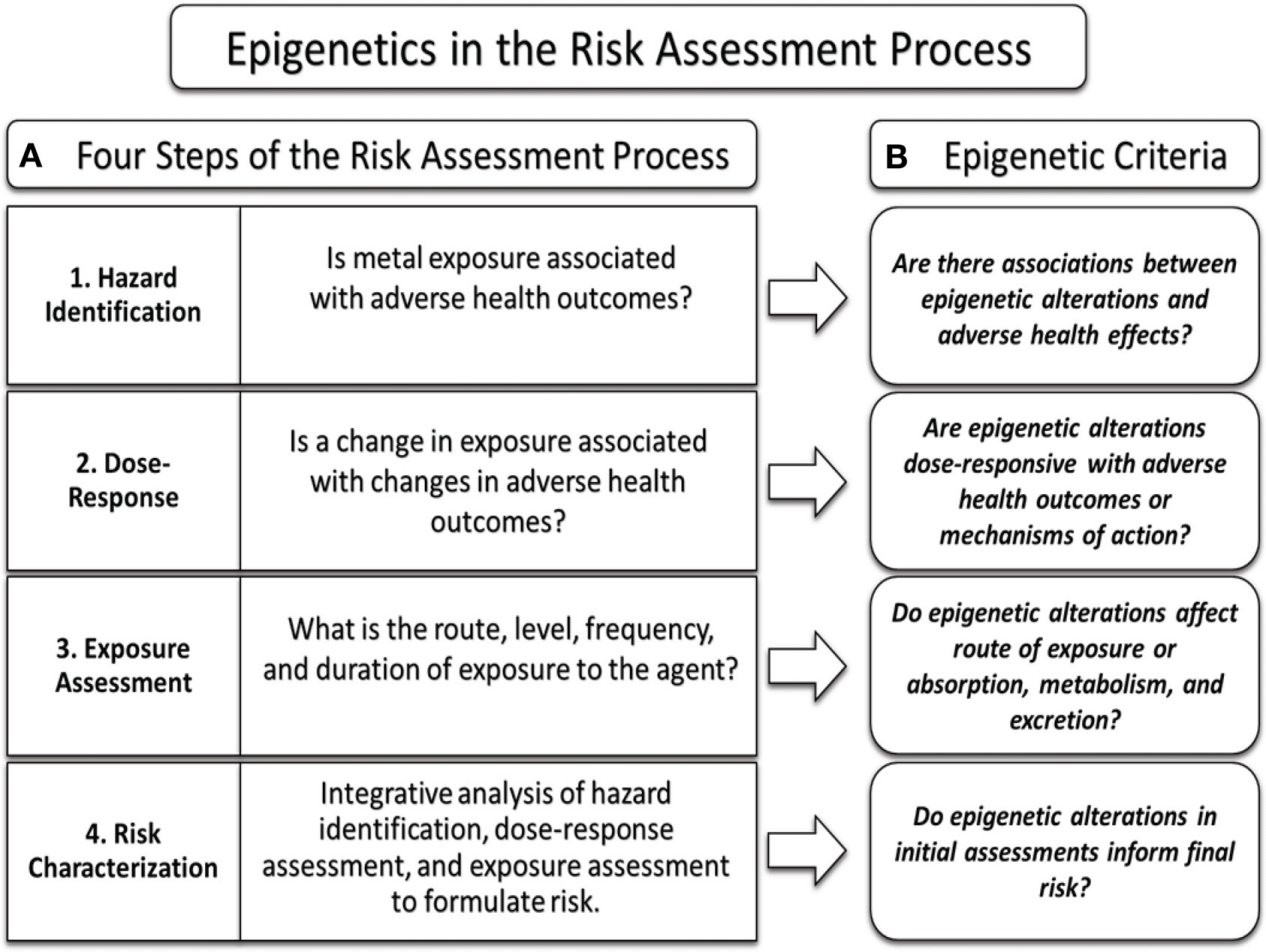
**Incorporating epigenetic data into the risk assessment process**. Epigenetic data may be used to inform each component of the risk assessment process. The risk assessment process consists of four key steps (**A**, left box); hazard identification, dose-response assessment, exposure assessment, and risk characterization. The intent of each step is met by applying clinical and epidemiological data to the criteria of each step (**A**, right box). Epigenetic data can be used to inform the risk assessment process by the integration of key data into the criteria framework **(B)** of each step in the process.

In the context of the risk assessment process (Figure 2), the first step, hazard identification, seeks to establish whether toxic-metal exposure is associated with a disease or mechanism of action leading to disease. Traditionally, toxicological studies have investigated exposure to toxic-metals and health outcomes, but due to the window of time between exposure and disease detection, epigenetic alterations may serve as better biomarkers linking historic metal exposure with disease. In the event that these epigenetic modification result in functional changes in gene expression and subsequent health outcomes, the epigenetic modification may serve to uncover a basis for metal-induced diseases where traditional toxicological studies have been limited.

Next, epigenetic alterations associated with toxic metals exposure may be used in the dose-response analysis, which seeks to determine if exposure is associated with health outcomes in a dose-dependent manner. If a dose-response relationship is identified between exposure, an epigenetic alteration, and functional gene expression leading to the disease, such epigenetic alterations may be able to inform the dose-response analysis.

The third step of the risk assessment process, exposure analysis, seeks to determine the route, degree, frequency, and duration of exposure for a particular agent. While studies have yet to substantiate toxic-metal induced epigenetic alterations as biomarkers of exposure, cord blood gene-specific expression data has been shown to be highly predictive of *in utero* arsenic exposure (Fry et al., 2007). If epigenetic alterations predict toxic metals exposure, such epigenetic data could be useful in informing the exposure components in the risk assessment process, and as an example, may supplement current arsenic biomarkers such as urine, blood, hair, or toenail samples (NRC, 1999).

Understanding the mechanism of action and mode of action of a particular agent in the disease process is a key component for the risk assessment process, and the use of epigenomic data may help to fill in current knowledge gaps. Associations between epigenetic alterations, gene expression, and disease initiation or progression may inform research into the modes of action of various toxic-metals. Additionally, epigenomic data has the potential to uncover whether toxic-metals may produce heritable epigenetic alterations. Ultimately such epigenomic data may directly play a role in the risk characterization step, and add additional accuracy to the risk assessment process.

### Epigenetic alterations as biomarkers of metals effects

There is the potential that epigenetic alterations can be used as biomarkers of adverse health outcomes, representing a new tool in predicting metal-associated diseases (Mulero-Navarro and Esteller, 2008; Bock, 2009). Stable epigenetic marks that are associated with a particular agent and adverse health outcome may also inform inter-individual differences in TK and TD. Additionally, as patterning of epigenetic marks naturally differ with the age of an individual (Calvanese et al., 2009), these data may be employed to estimate if exposure occurred during sensitive windows of development (pre- or post-fertilization, *in utero*, or puberty), which are associated with varying health outcomes. Such biomarkers associated with disease may be used as an additional tool that could inform the risk assessment process.

In the clinical settings, epigenetic biomarkers may be useful as early detection of changes associated with chronic diseases or cancers. For example, epigenetic alterations associated with tumor suppressors or oncogenes may inform an individual's cancer susceptibility (Verma et al., 2003). Specific epigenetic marks on such genes can be associated with the stage of cancer development, thus informing diagnosis and treatment. These techniques have the possibility of being applied to metal-induced carcinogenesis.

Clearly, additional studies are needed to ascertain metal-associated epigenetic alterations not only at different exposure levels, but also at varied developmental time points. Such information is critical not only to refine dose-response predictions, but to differentiate between epigenetic alterations that naturally differ with respect to age (Bocklandt et al., 2011), and those associated with toxic-metal exposure. Additionally, the focus on these changes would need to be shown to be exposure specific and independent of other confounding agents. Such research would need to also assess whether such epigenetic marks were loci and tissue specific, and whether such biomarkers were representative across populations.

### Epigenetic alterations as predictors of metal-induced gene expression

Epigenetic alternations may be a useful tool in predicting differential gene expression in response to toxic-metal exposure. As mentioned previously, epigenetic alterations can affect gene expression in a number of ways, including histone modifications which influence transcription, expression of miRNAs which regulate mRNA stability and subsequent translation, and modification of CpG methylation patterns leading to altered transcription. Of the three, alteration of DNA methylation patterns is supported by the largest body of evidence. Methylation of CpG sites in the promoter regions of genes is often associated with silencing the expression of that gene (Weber et al., 2007). While many studies have shown a global inverse association between methylation and expression, it is worth noting that such an association is not universal (Bell et al., 2011), and as such epigenetic data should be complemented with corresponding gene expression data. There is the potential that some epigenetic marks may enable researchers to predict gene specific expression changes and their associated health outcomes.

### Epigenetic alterations as predictors of toxicity and disease

The potential for heritability associated with forms of epigenetic alterations provides novel possibilities in the risk assessment process. Epigenetic modifications can be mitotically inherited, and as a result may be passed on to future generations (Rakyan and Whitelaw, 2003). As a result, heritable epigenetic alterations may play a pivotal role in determining inter-individual variation in susceptibility to disease based on the differences in individual's ancestral environmental exposures. Because epigenetic reprogramming is utilized by the body as a mechanism to ensure proper cell development and function (Reik et al., 2001), disruption of these patterns during key developmental windows can produce stable phenotypic and functional changes in an individual (Tang and Ho, 2007).

As discussed in the review, *in utero* exposure to several toxic metals such as arsenic and cadmium are associated with epigenetic alterations. Epigenetic alterations not only provide the possibility of informing later life health outcomes, but may be useful in predicting adverse health outcomes from a specific early life metal exposure. Inter-individual differences in metabolism may actually be mediated in part by the epigenetic modifications. In certain cases, metal-specific epigenetic alterations may have the possibility of informing researchers of an individual's previous exposure and their specific associated changes in gene expression or health effects.

In addition, epigenetic alterations to the germ line may provide insight into trans-generational differences in disease patterns or susceptibility (Sasaki and Matsui, 2008; Skinner, 2008). Individuals in the F3 generation, with no direct contact to the originally exposed F0 ancestor may still experience functional changes as a result of their ancestor's historical environment. While such a concept has yet to be shown in humans exposed to toxic-metals, researchers have shown exposure to famine is associated with sex-specific mortality differences in an individual's grandchildren (Heijmans et al., 2008). Additionally, research in rodent models has shown differences in physiology and cancer susceptibility persist after four generations in rats originally exposed to various endocrine disruptors (Anway et al., 2005). While the transgenerational nature of epigenetic alterations is under debate, it is worth noting such a phenomenon could greatly inform the risk assessment process if heritable epigenetic alterations associated with disease can inform intra-individual differences in disease susceptibility.

## Final considerations

While the number of publications on metals-induced epigenetic changes continues to rise, there are gaps in research that need to be filled prior to their incorporation into the risk assessment process. Where data are derived from humans, sample sizes need to be large and data confirmed and replicated cross-cohorts. The data should be collected at different time points during development and/or growth to determine the temporal stability of the changes and establish potential developmental windows of susceptibility. Moreover, additional studies are required to examine the associations between epigenetic alterations, functional cellular consequences and adverse health outcomes through the use of epidemiologic as well as traditional toxicological studies. Such studies require an examination across multiple tissues, at multiple doses, for multiple durations, and at different time periods during development or life stage.

The epigenome serves as a link between an individual's genome and their response to environmental cues. As such, the changes to the epigenome can act as both a snapshot of an individual's response to various environmental stressors, as well as a putative predictor of an individual's susceptibility to future stressors and disease outcome. In order to fully understand these relationships it will be necessary to conduct studies that compare both epigenetic data with functional measures such as gene and protein expression within the same study and controlled exposure scenarios. Taken together, the epigenetic data may provide novel contributions to the risk assessment process, but currently much remains unknown about the association between these modifications and functional changes in gene and protein expression.

### Conflict of interest statement

The authors declare that the research was conducted in the absence of any commercial or financial relationships that could be construed as a potential conflict of interest.
